# Nature-based climate solutions require a mix of socioeconomic and governance attributes

**DOI:** 10.1016/j.isci.2022.105699

**Published:** 2022-11-30

**Authors:** Ernest F. Asamoah, Joseph M. Maina

**Affiliations:** 1 School of Natural Sciences, Macquarie University, North Ryde 2109, NSW, Australia

**Keywords:** Earth sciences, Earth-surface processes, Environmental science, Social sciences

## Abstract

Nature-based climate solutions (NCS) can play a crucial role in reducing climate change. There is, however, a lack of understanding of the biophysical, social, and political contexts surrounding NCS, which hampers its practical implementation. Here, we used estimates of carbon sink potential to identify socioeconomic and ecological factors that may stimulate NCS implementation in developing economies. We considered carbon sink potential for eight NCS, including reforestation, peatland restoration, natural forest management, improved rice cultivation, optimal grazing intensity, grazing (legumes), avoided peatland impacts, and avoided coastal impacts. Food insecurity hotspots, which currently receive the most development aid, have the lowest likelihood of realizing NCS’ potential. Poor governance structures and food insecurity impede the implementation of NCS projects at the country level. By carefully assessing complementary food security, sustainable financing, and soil quality safeguards, NCS as a nationally determined contribution to climate mitigation can be made more effective.

## Highlights

• Harnessing nature’s potential can bolster climate mitigation efforts
• Food insecurity and poor governance can constrain these benefits
• Sustainable financing and food security safeguards can boost NCS adoption

## Graphical abstract

**Figure:**
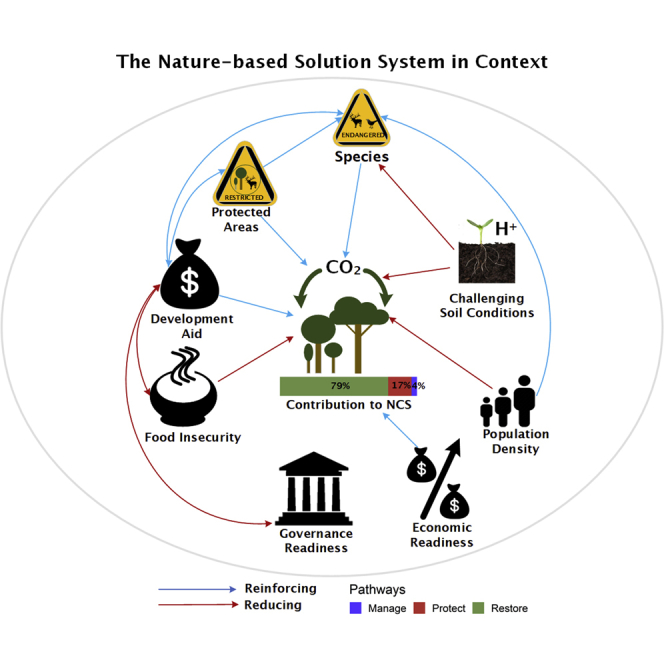

## Introduction

Protecting, restoring or managing natural and semi-natural ecosystems can deliver multiple benefits to people and nature.^1^^,^^2^ These options, collectively conceived as NCS, are critical transitions and cost-effective approaches for a sustainable and resilient land-use system to control climate change.^3^^,^^4^^,^^5^^,^^6^ Indeed, if properly implemented, NCS can contribute over 30% (by 2030) to carbon uptake globally,^4^^,^^5^ and could reduce the global peak temperature by 0.3°C under a 2°C warming trajectory by 2085 (or by 0.1°C by 2055 under a 1.5°C rise).^7^ Along with climate change mitigation, NCS offers benefits for biodiversity, human well-being, water purification and soil protection.^8^^,^^9^^,^^10^

Amid the frantic efforts to combat climate change and biodiversity loss, NCS is garnering global and local attention, primarily because of their demonstrated positive role in climate change and biodiversity loss mitigation across multiple biomes. Recent studies have mapped and identified opportunities for restoring forest cover and forest protection as NCS at local, national and global scales.^11^^,^^12^^,^^13^ Despite the increased focus on NCS and on identifying the accrued benefits, there is a lack of systematic analysis of the biophysical, social and political contexts of their uptake and effectiveness.^10^ Fundamental concerns are that widespread climate change-induced die-offs of forests during the 21st century may compromise forest carbon stocks and sinks^14^ and influence natural regeneration.^13^ Landscape legacies of management activities and human impacts occurring today, such as agriculture expansion, infrastructural development and area-based management^15^^,^^16^^,^^17^^,^ can promote future adaptation by generating present capabilities and benefits—or may become an impediment to transformation.^10^ Among the factors that can impede successful NCS implementation, financial constraints can reduce the climate mitigation potential of NCS in most parts of the world—for example, in Southeast Asia by ∼55% (low estimate) to ∼17% (high estimate).^18^ In addition, harnessing nature to slow climate change may create land allocation issues, exacerbate food insecurity and widen social inequities already strained by climate change and wars.^19^^,^^20^ Mainstreaming climate change mitigation and biodiversity conservation actions at the global to local levels is contingent on knowledge, institutional capacity and public participation.^5^^,^^21^^,^^22^ Understanding the factors that promote NCS is a vital first step to ensuring long-term increases in forest cover, carbon storage and biodiversity.^3^^,^^20^

Here, we aim to provide this understanding by testing for the association between contemporary socioeconomic factors and the potential carbon sink from management, restoration and protection efforts (hereafter NCS) across 126 nations with ‘developing economies’ status. Empirical research on the biophysical and social contexts of NCS, including their co-benefits, is necessary for addressing the existing gap in policy-relevant science on socioeconomic and ecological factors that may limit the implementation of NCS, including synergies for maximizing ecosystem services.^9^^,^^23^^,^^24^ A recent empirical study shows that the cost-effective potential of NCS is lowest in regions with feasibility concerns.^25^ Whereas cumulative feasibility can inform the development of spatial NCS policies, assessing individual feasibility constraints to NCS will likely unravel synergies that can inform the integration of policies for reducing emissions, biodiversity loss and improving well-being, commonly viewed as separate policy concerns.^26^^,^^27^ Therefore, we conceptualize NCS and socioecological and economic factors in a structural framework depicting NCS within a more holistic socio-ecological system space^3^ using nature-based thinking.^28^ Applying statistical methods to future biophysical estimates and contemporary socioeconomic indices can help determine potential regions, but socioeconomic factors may catalyze the design and deployment of NCS projects (see also Boyce et al.^29^). In practice, NCS interventions – particularly those targeted toward enhancing carbon stocks and promoting sustainable forest management – seldom consider co-benefits.^30^

## Results and discussion

### Climate mitigation potential from NCS

**Figure 1:**
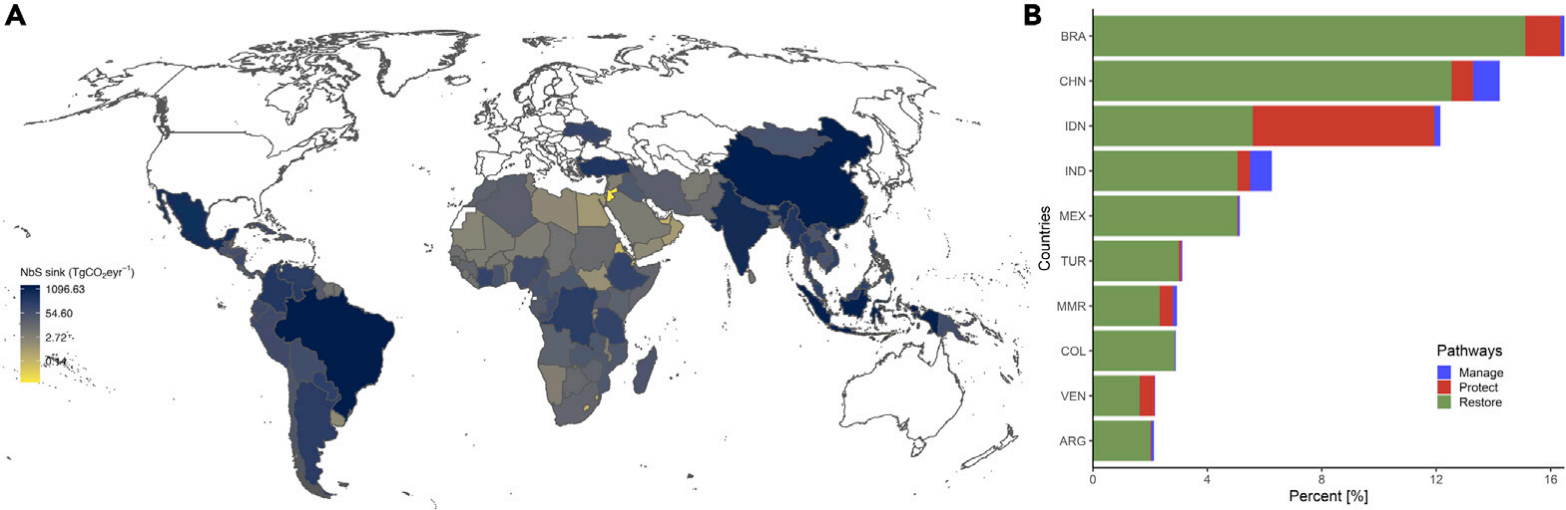
Carbon-sink potential sink potential for nature-based climate solutions (A and B) Maximum carbon-sink potential from management, protection, and restoration actions across developing countries, and (B) ten nations where NCS potential is highest grouped by the three main NCS pathways: (1) Restoration, (2) protection and (3) management. Administrative boundary of map elements is based on www.gdam.org (v3.6). International Standards Organization (ISO) alpha-3 country codes indicates: BRA = Brazil, CHN = P.R. China, IDN = Indonesia, IND = India, MEX = Mexico, TUR = Turkey, MMR = Myanmar, COL = Columbia, VEN = Venezuela, and ARG = Argentina. See also Table S1 and Figure S2.

Climate mitigation potential from implementing eight NCS across 126 developing countries worldwide is ∼10.3 PgCO_2_yr^−1^, representing ∼42% of the global NCS,^4^ and included 33% of restoration, 7% of protection, and 2% management pathways (Figures 1A and 1B). The five developing countries with the potential for NCS to accumulate the greatest amount of carbon are Brazil (1700.5 TgCO_2_yr^−1^), China (1466.7 TgCO_2_yr^−1^), Indonesia (1252.8 TgCO_2_yr^−1^), India (645.2 TgCO_2_yr^−1^) and Mexico (529.4 TgCO_2_yr^−1^) [Figure 1B]. These countries retained 54% of the maximum annual climate mitigation potential from the eight NCS considered in the analyses. NCS also differed across regions; a higher proportion of mitigation potential is estimated in Asia (46%) compared to Latin America (38%), Africa (12%) and Europe (4%) (Figure 1, Table S1).

### NCS within a socio-economic system

**Figure 2:**
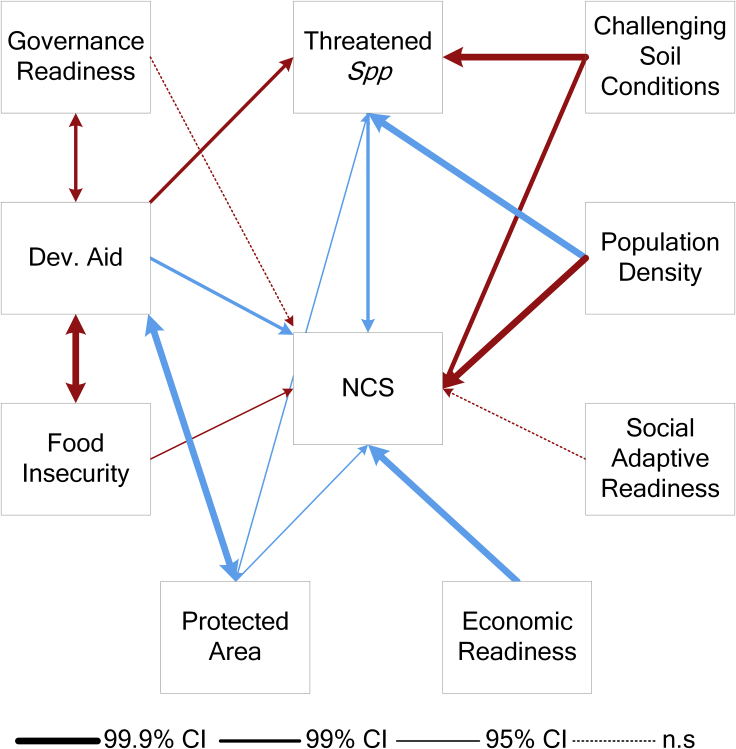
NCS within a socio-ecological system Path width corresponds to the degree of significance of the associations, as shown in the legend. Blue and red arrows indicate positive and negative associations, respectively. Dashed lines indicate non-significant associations (n.s., p> 0.05). Double arrows indicate covariance. See also Figure S2. Dev. Aid refers to development aid.

Using 11 socioecological factors, we generated all possible combinations before testing for collinearity using variance inflation factor (VIF) tests using the *usdm* package in R.^31^^,^^32^ Model constructs with a VIF > threshold of 1.5 were discarded. Consequently, 399 model constructs were fit using ordinary least squares (OLS) with NCS as the response in a model selection framework. The best-selected models (n = 5, ΔAICc<2, Table S4) comprised nine socioeconomic and ecological factors and explained 72% of the distribution of NCS. The best models included: efforts and effectiveness (i.e., development aid, governance readiness, adaptive social readiness and protected areas coverage), social status (food insecurity, population density and national-level gross domestic product [national GDP]), and legacy of recent change and ecological conditions (climate, land-use, topsoil pH and threatened tetrapods). Moreover, conceptualizing NCS and top model variables within a socio-ecological system space^3^ showed linkages that constitute additional pathways that can complement the design of NCS projects. For example, additional pathways essential for prioritizing global conservation spending, threatened biodiversity, food insecurity, and the size of the area to be conserved^33^ were also strong in the system (Figure 2).

Uptake and design are among the key components of the NCS framework.^34^ NCS positively correlates with protected areas coverage, indicating a disproportionately greater NCS for regions that have made progress toward fulfilling international commitments. The overall effectiveness of protected areas as a cost-effective nature-based tool is contingent on governance structures that balance the tradeoffs between climate mitigation and societal menaces of land protection.^35^^,^^36^ Moreover, we estimated a greater NCS for regions that received relatively greater development aid, suggesting the current distribution of development aid is appropriately targeted in regions where land management, protection, and restoration NCS is greatest. The positive association between aid and NCS could attenuate in poorly-governed countries, implying that ineffective governance structures may hinder NCS-oriented funds allocation. Indeed, trust in the program being undertaken influences multilateral consortias’ willingness to provide capital support.^3^ Previous empirical reporting has suggested that good governance increases conservation aid and that performance-oriented projects work in better-governed regions.^37^^,^^38^

Effective governance and improved social capacity are essential to ensure NCS addresses the needs of people and nature.^2^ However, indicators of governance and adaptive social readiness are not strong correlates of NCS on their own, suggesting no strong evidence that effective governance and improved social capacity foster a good environment for the design and deployment of NCS projects. Infrastructure deficit across developing countries may explain the weak association between social adaptive readiness and NCS. The lack of strong evidence of governance and environmental performance has been reported elsewhere.^39^

**Figure 3:**
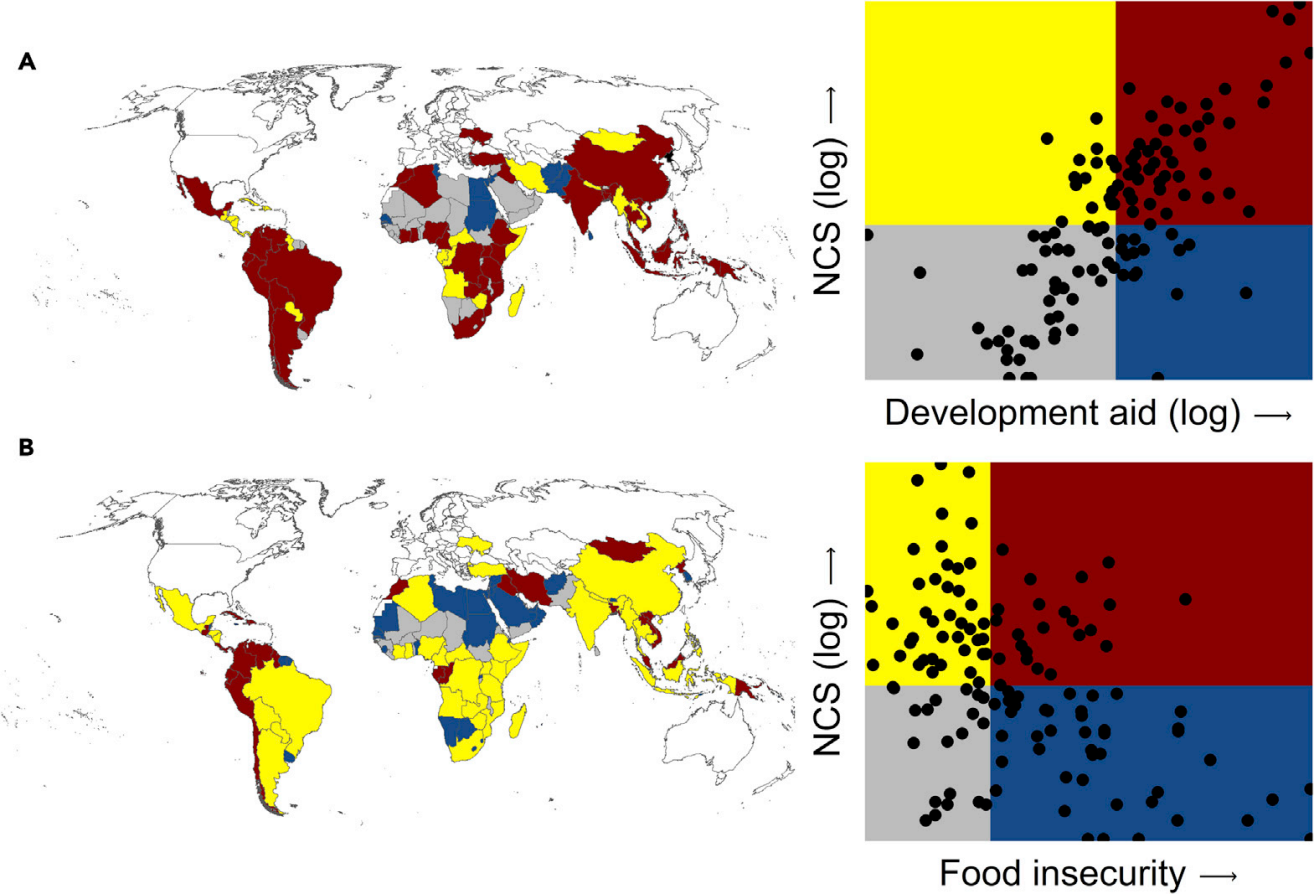
Overlap of NCS and some key factors Bivariate choropleth maps of NCS and social indicators; (A) development aid and (B) food insecurity, are divided into quartile bins, and included including low NCS potential and low development aid or food insecurity (gray shades), high NCS and low development aid or food insecurity (yellow shades), high NCS and high development aid or food insecurity (red shades), and low NCS and high-development aid or food insecurity (blue shades). Regions with a white background are developed nations, transitioning economies, or those missing one or more variables. The administrative boundary of maps is based on www.gdam.org (v3.6). See also Table S3.

Moreover, we found a greater NCS are estimated for more food-secure regions of the developing world (Figure 3). These regions are located predominantly in North Africa and East Asia and include densely populated but wealthier developing nations (Figure 3B). This implies that uptake of NCS may decline disproportionately in densely populated regions where food production and supply patterns are highly variable. By considering the main NCS pathways separately and assessing nonlinearities, a more nuanced understanding of how the uptake and design of NCS are influenced by sociological-ecological factors emerged (Figure S1). For example, restoration NCS appears to be disproportionally higher in densely populated regions, unlike protection and management pathways. From a feasibility perspective, restoration NCS may require shifting land use, which can face a host of cultural, social and economic barrier.^40^

A disproportionately greater potential estimated for developing nations (higher national GDP) with relatively higher numbers of threatened tetrapods indicates higher restoration, protection and management potential in regions where biodiversity safeguards are paramount.^41^ A negative association was found between NCS and topsoil pH: greater NCS are estimated disproportionately for regions with soil nutrient availability challenges. This finding underscores the importance of soil quality improvements in NCS discussions, although additional efforts are required to increase the feasibility of management strategies.^42^ Soils are one of the important sinks for carbon globally, and soil quality safeguards will likely be key in hotspots. This finding is significant for the restoration or reforestation of NCS pathways where seedlings’ regeneration, growth and survival are considered imperative.^43^ Nationally determined contributions (NDCs) associated with reforestation and restoration should include land-management practices to increase the resilience of managed lands to future climate extremes (for example, nature-based agriculture [i.e., agroforestry]). Using carefully selected exotic or native trees for rehabilitating degraded soils can improve soil fertility and enhance biodiversity.^44^ The differential contributions of topsoil pH to NCS through different pathways demonstrate land management’s positive role in nutrient improvement co-benefits. Therefore, an integrated strategy could better reduce soil losses and improve soil quality than strategies applied individually.^42^

### Implications for sustainable nature-based solutions

Meeting the goals of the Paris Agreement of keeping global warming below 2°C will require, among other strategies, ‘natural’ methods such as land management, restoration and protected areas.^6^ Socioeconomic factors may likely constrain their update, design and success. Here, relating socioeconomic and ecological factors to ecosystem performance under the broader umbrella of nature-based solution progress our understanding of social security, sustainable financing, and governance implications for controlling global environmental changes. However, our study uses statistical extrapolations that assume underlying processes will remain constant in the future. Uncertainties in estimating carbon mitigation and assumptions surrounding statistical extrapolations are noteworthy caveats of our study. Moreover, NCS are more than carbon uptake, and focusing only on land-based mitigation potential limits the assessment of NCS overall. Owing to data availability, we have focused on only eight NCS pathways, which globally is approximately half of the 20 original pathways. Despite these limitations, our study identified socioeconomic factors likely to constrain the uptake, design, and success of NCS as a nationally determined contribution to climate change mitigation.

Evolving global policy frameworks, including the CBD’s proposed post-2020 draft biodiversity framework, underscore the need to concurrently mitigate climate change, conserve biodiversity and enhance human well-being.^45^ NCS provides a roadmap to sustainability and benefit-sharing at multiple spatial scales, which likely will strengthen the first three Sustainable Development Goals (SDGs 1, 2, 3)—No Poverty, Zero Hunger, and Good Health and Wellbeing—especially in developing nations. Our analyses suggest that, with the projected exponential growth of populations,^46^ developing countries may face difficulties implementing internationally agreed commitments and making trade-offs between food security needs and NCS activities. Concerns over the potential impact of surging food prices on future carbon sequestration services of NCS have been raised elsewhere.^19^ Careful consideration of population growth is needed when developing strategies to achieve internationally agreed climate change mitigation commitments. A sustainable socio-ecological design of NCS would require government, local authorities, community-based organizations, private-sector initiatives and impact investors to focus on landscape and payments for ecosystem services schemes to define their desired outcomes and target management actions accordingly clearly.

A lack of sufficient and sustainable funding and incentives is one of the main barriers to implementing and monitoring NCS worldwide.^2^^,^^3^ Given that NCS is gaining traction within the sustainable development discourse, with the development of several frameworks (for example, the CBD’s zero-draft post-2020 global biodiversity framework and the SDGs), policymakers and multilateral and bilateral funding agencies will require scientific evidence on allocating funding for NCS activities. We observed that the current distribution of biodiversity conservation and social wellbeing funding is appropriately targeted in regions with potential. Nonetheless, currently, there appears to be a huge deficit in investments specifically targeted to NCS activities, with only US$52 billion of the required US$300–400 billion per year investments being invested into ecosystem preservation and restoration projects.^2^ Whether the current flow of funding can support the adoption and design of NCS will depend on sustainable financing and governance of conservation funds.^3^ The reduced association between development aid and NCS for poorly governed nations illustrate these potential governance challenges. They could further suggest that ineffective governance may make NCS unattractive to multilateral donors and funders. There is a need to track and monitor progress if the current funding trajectory is to be adopted. Examples of successful implementation of NCS demonstrate that effective NCS occur at a micro-scale, often in landscapes where local communities and Indigenous peoples live. Consequently, careful consideration of cooperation between national governments, local authorities and impact investors is required to foster successful socio-ecological design and deployment of NCS.

It is important to recognize that delayed implementation will likely reduce the cost-effectiveness of NCS by 2030^3^^,^^4^ and that urgent action is, therefore, necessary.^47^ Our findings indicate additional pathways for NCS allocation and design, elucidating the interplay of policies aimed at emission reduction and those targeting biodiversity conservation. For instance, it is essential to approach and prioritize regions for reducing emissions from deforestation and forest degradation plus (REDD + targeting) under budget constraints. As our study illustrates, good governance is indispensable for the success of NCS initiatives, and better land stewardship can promote and transform national economies and contribute to achieving NDCs.

In conclusion, our study identified socioeconomic and ecological correlates of the uptake and design of NCS. These associations differed markedly among restoration, protection and management NCS pathways, which can inform land-use decisions made at local and national scales. The observed direct and indirect associations suggest that one important new way of thinking about ecosystem futures is considering the interactions between multiple interventions. Our findings underpin NCS pathways as strategies to achieve numerous goals beyond climate mitigation, including biodiversity conservation, socioeconomic benefits, food security and ecosystem services.

## STAR★Methods

### Key resources table

**Table:** 

REGEANT or RESOURCE	SOURCE	IDENTIFIER
**Depositeddata**		

Carbon mitigation potential	Griscom et al.^4^	https://www.pnas.org/doi/10.1073/pnas.1710465114#supplementary-materials
ND-GAIN Database	ND-GAIN ^48^	https://gain.nd.edu/our-work/country-index/
World Bank Geocoded Aid Data v1.4.2	AidData^49^.	https://www.aiddata.org/data/world-bank-geocoded-research-release-level-1-v1-4-2
WDPA Database	UNEP-WCMC and IUCN^50^	https://www.protectedplanet.net/en/thematic-areas/wdpa?tab=WDPA
FAO’s Food Security Indicators	NA	https://www.fao.org/food-agriculture-statistics/en/#.XzibF6fiuHs
LANDSCAN	Roe et al. ^51^	https://landscan.ornl.gov/
World Development Indicators	NA	http://wdi.worldbank.org/
WorldClim (v2)	Fick and Hijman ^52^	http://www.worldclim.com/version2
Topsoil pH	Fischer et al. ^53^	https://www.fao.org/soils-portal/data-hub/soil-maps-and-databases/harmonized-world-soil-database-v12/en/
IUCN RedList database	IUCN ^54^	https://www.iucnredlist.org/
GADM database (v3.6)	GADM ^55^	https://gadm.org/download_world36.html

**Software andalgorithms**		

*Usdm:*Uncertainty Analysis for Species Distribution Models	Babak et al.^31^	https://cran.r-project.org/web/packages/usdm/index.html
R statistical platform	R Core Team^32^	https://www.r-project.org/
*piecewiseSEM:*Piece-wise Structural Equations Modeling	Lefcheck et al. ^56^	https://cran.r-project.org/web/packages/piecewiseSEM/index.html
*Gbm:*Gradient Boosting Machines	Elith et al.^57^	https://cran.r-project.org/web/packages/gbm/index.html

### Resource availability

#### Lead contact

Further information and requests should be directed to and will be fulfilled by the lead contact, Ernest F. Asamoah asamfrt@gmail.com.

#### Materials availability

This study did not generate new unique reagents.

### Experimental model and subject details

Our goal was to examine associations between socioecological factors and land-based NCS for climate change mitigation across 126 nations of developing economic status.

### Method details

#### Carbon sink from NCS

We first accessed existing datasets on carbon sinks potential (TgCO_2_yr^−1^).^4^ A total carbon sink is defined as the maximum carbon mitigation potential above a business-*as*-usual baseline at a reference year. We focused on eight available NCS, including reforestation, natural forest management, improved rice cultivation, grazing (optimal intensity), grazing (legumes), peatland restoration, avoided peatland impacts, and avoided coastal consequences (mangroves). To distinguish between major land stewardship approaches, the total NCS was re-categorised into restoration, protection, and management pathways.

We then hypothesised that socioeconomic and ecological factors correlate with NCS. These factors are represented using 11 variables broadly placed into three categories: Efforts and effectiveness, comprising governance readiness ^48^ , social adaptive readiness ^48^ development aid ^49^ and preotecteds areas ^50^; Social statuses, comprising food insecurity, population density ^51^ and economic readiness; and legacies of recent land-use climate and ecological attributes, containing, climate ^52^, topsoil pH ^53^, and richness of threatened tetrapods ^54^ (see STAR Methods table, Table S5). The quality of governance is key to shaping which interventions are adopted and why. It is essential to understand how financing, implementation and governance of those interventions are important components of the NCS framework.^3^ Effective governance and improved social capacity will foster an adequate environment for the deployment and subsequent performance of NCS. Both metrics were averaged between 2000 and 2012 for each country. Governance readiness is a composite of political stability and non-violence, control of corruption, the rule of law, and regulatory quality. Adaptive social readiness encapsulates social inequality, ICT infrastructure, education and innovation. Funding is essential for nations and local communities to build resilience to climate change. Indeed, economically sound (sustainable) financing approaches can foster the decarbonisation of the global economy and enhance biodiversity conservation.^3^^,^^58^ To represent funding, we retrieved development aid data from Aid database ^48^.

### Quantification and statistical analysis

#### Statistical analysis

We use multiple linear regression to test the association between carbon sink (a proxy for climate mitigation potential from NCS) and 11 socioeconomic and ecological factors. Regression models were based on normality and residual independence assumptions of ordinary least squares (OLS) regression. Initial data assessments revealed multi-collinearity among explanatory variables—for example, absolute Spearman’s rho (|*ρ*|) ranged between 0 and 0.78 and the maximum variance inflation factor (VIF) was 11.89 (Tables S2 and S3). To reduce the effects of collinearity on estimates, candidate model combinations were filtered using a threshold VIF of less than 1.5 (see McClanahan et al.^59^). All possible regression-model combinations of fixed factors that passed the collinearity threshold (n = 399) were compared for their fit to the data using order second-order Akaike’s Information Criterion (AICc) and ranked serially (Table S4). We selected the best-fitting models within ΔAICc<2. Estimates for fixed factors shown are conditional averages between best-selected models (Table S5).

Next, we fitted structural equation models to examine indirect associations among factors retained in the model selection approaches. We use SEM to test for non-mutually exclusive associations among each system’s components. ^60^ Our SEM framework includes two major interrelated components: NCS sink and socioeconomic and ecological factors. Because different socioeconomic and ecological factors might be interrelated, linkages may constitute additional pathways by which environmental and socioeconomic factors might influence NCS. For example, four primary considerations are essential for prioritising global conservation spending: threatened biodiversity, cost, cost-effectiveness (likelihood of success) and the size of the area to be conserved.^33^ That is, donors are likely to fundNCS when projects can provide co-benefits at the least cost of implementation. We fit SEM using *piecewiseSEM* (v2.1.0), which is known to overcome distributional and data independence limitations of traditional variance-covariance SEM.^32^^,^^56^^,^

To ensure that all regression assumptions were met, numerical covariates were transformed to normality using Tukey’s ladder of powers where necessary. Additionally, data for numerical covariates were scaled by subtracting the mean and dividing by one standard deviation (SD) to interpret coefficients. Associations between sink and factors appeared complex and exhibited a non-gaussian behavior. We relaxed OLS constraints and distributional assumptions and fitted boosted regression trees to the data to complement the analysis. We chose optimal combinations of tree complexity, learning rate and bag fraction that minimise out-of-bag estimates of error rates.^57^

## Data Availability

Datasets used to support the findings of this study are freely available online (sources are supplied in resource tables). This paper does not develop original codes. Any additional information required to reanalyze the data reported in this paper is available from the lead contact upon request.
