# Supplementary material for: Nature-based climate solutions require a mix of socioeconomic and governance attributes
Source: iScience. 2022 Nov 30;25(12):105699. doi: 10.1016/j.isci.2022.105699 (PMC9768352; doi:10.1016/j.isci.2022.105699)
Supplement: Document S1.Figures S1, S2 and Tables S1–S5 [file mmc1.pdf]

iScience, Volume *25*

Supplemental information

Nature-based climate solutions require a mix of socioeconomic and governance attributes

Ernest F. Asamoah and Joseph M. Maina

Table S1. Regional variation of NCS, related to Figure 1

Continent	NCS ($TgCO_2yr^{-1}$)	Proportion (%)
Asia	4713.12	46.0
Latin America	3878.41	37.8
Africa	1221.75	11.9
Europe	436.99	4.3
**Total**	10250.27	

Table S2. Correlation matrix (Spearman's rank correlation) of eleven potential correlates of the sink. Variance inflation factor (VIF) for explanatory factors. VIF1 and VIF2 refer to the initial and final VIFs, respectively. X: variables not selected as part of top models. *Variables are transformed to normality using Tukey's ladder of powers, which finds the power transformation which maximises normality as assessed by Shapiro–Wilkinson tests. Strong correlations (Spearman's rho |ρ| > 0.7) are shown in bold. **Related to STAR Methods**.

		**v1**	**v2**	**v3**	**v4**	**v5**	**v6**	**v7**	**v8**	**v9**	VIF1	**VIF2**
**v1**	Population density*										7.56	2.44
**v2**	PA coverage*	−0.29									3.22	3.13
**v3**	National GDP*	0.29	0.50								9.35	4.45
**v4**	Food insecurity	−0.11	−0.30	−0.16							2.17	1.94
**v5**	Development aid*	0.34	0.39	**0.78**	−0.37						4.78	3.92
**v6**	Governance readiness	−0.12	0.01	0.02	0.53	−0.22					2.07	1.65
**v7**	Social readiness	0.08	0.09	0.38	0.14	0.28	0.09				1.36	1.31
**v8**	Threatened tetrapods*	0.28	0.09	−0.09	−0.17	−0.04	−0.13	−0.13			2.57	2.53
**v9**	Climate variability	−0.11	0.07	0.13	0.30	0.08	0.28	0.16	−0.10		1.36	x
**V10**	Topsoil pH	−0.05	−0.06	0.17	0.15	0.05	0.10	0.15	**−0.72**	0.08	2.27	2.25

Table S3. Top 10 models of 399 ensemble combinations of variables explaining the distribution of sink. v1 = Climate variability, v2 = food insecurity, v3 = social readiness, v4 = topsoil pH, v5 = PA coverage, v6 = population density, v7 = governance readiness, v8 = national gross domestic product (GDP), v10 = development aid. Models with second-order Akaike Information Criterion of less than two (AICc<2) are shown in bold. **Related to STAR Methods**.

v1	v2	v3	v4	v5	v6	v7	v8	v9	v10	AICc	delta
	**x**		**x**		**x**			**x**		**–149.255**	**0.000**
	**x**				**x**	**x**	**x**	**x**		**–148.652**	**0.603**
	**x**	**x**	**x**		**x**			**x**		**–148.408**	**0.847**
	**x**				**x**	**x**		**x**		**–147.720**	**1.535**
				**x**		**x**			**x**	**–147.441**	**1.814**
			x	x					x	–147.053	2.202
x	x		x		x			x		–147.044	2.211
	x	x			x	x		x		–146.487	2.768
x	x	x	x		x			x		–146.206	3.049
x	x				x	x		x		–145.422	3.833

Table S4. Model-average results of multivariate models (that is, 5 of 399 ensemble model combinations) explaining NCS. **Related to Figure 3 and STAR Methods.**

Explanatory factors	Estimate	Std. Error	Adjusted SE	z value	Pr(>|z|)
(Intercept)	0.629	0.483	0.483	1.300	1.93E-01
National GDP	0.843	0.076	0.077	10.961	0
Development aid	0.589	0.079	0.080	7.343	0
PA coverage	0.676	0.098	0.099	6.836	0
Topsoil pH	-0.083	0.012	0.013	6.566	0
Threatened tetrapods	0.371	0.060	0.060	6.136	0
Food insecurity	–0.850	0.155	0.156	5.434	6.00E-08
Population density	–0.381	0.098	0.099	3.835	1.25E-04
Governance readiness	–0.191	0.108	0.110	1.741	8.17E-02
Adaptive social readiness	–0.216	0.182	0.184	1.171	2.42E-01

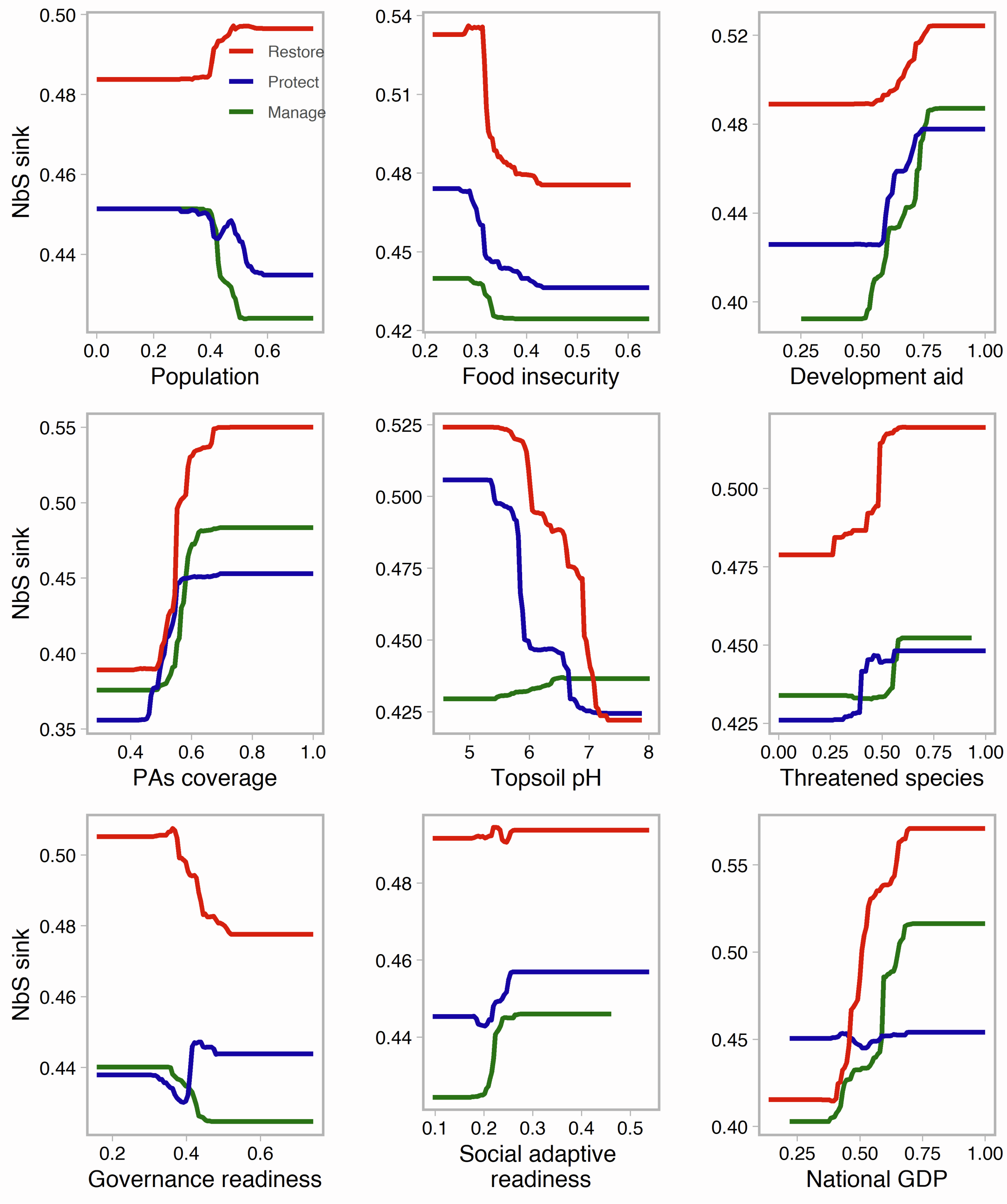

Figure S1. Partial dependence plot of boosted regression tree estimating associations between of socioeconomic and ecological factors on NCS. Related to Figure 2.

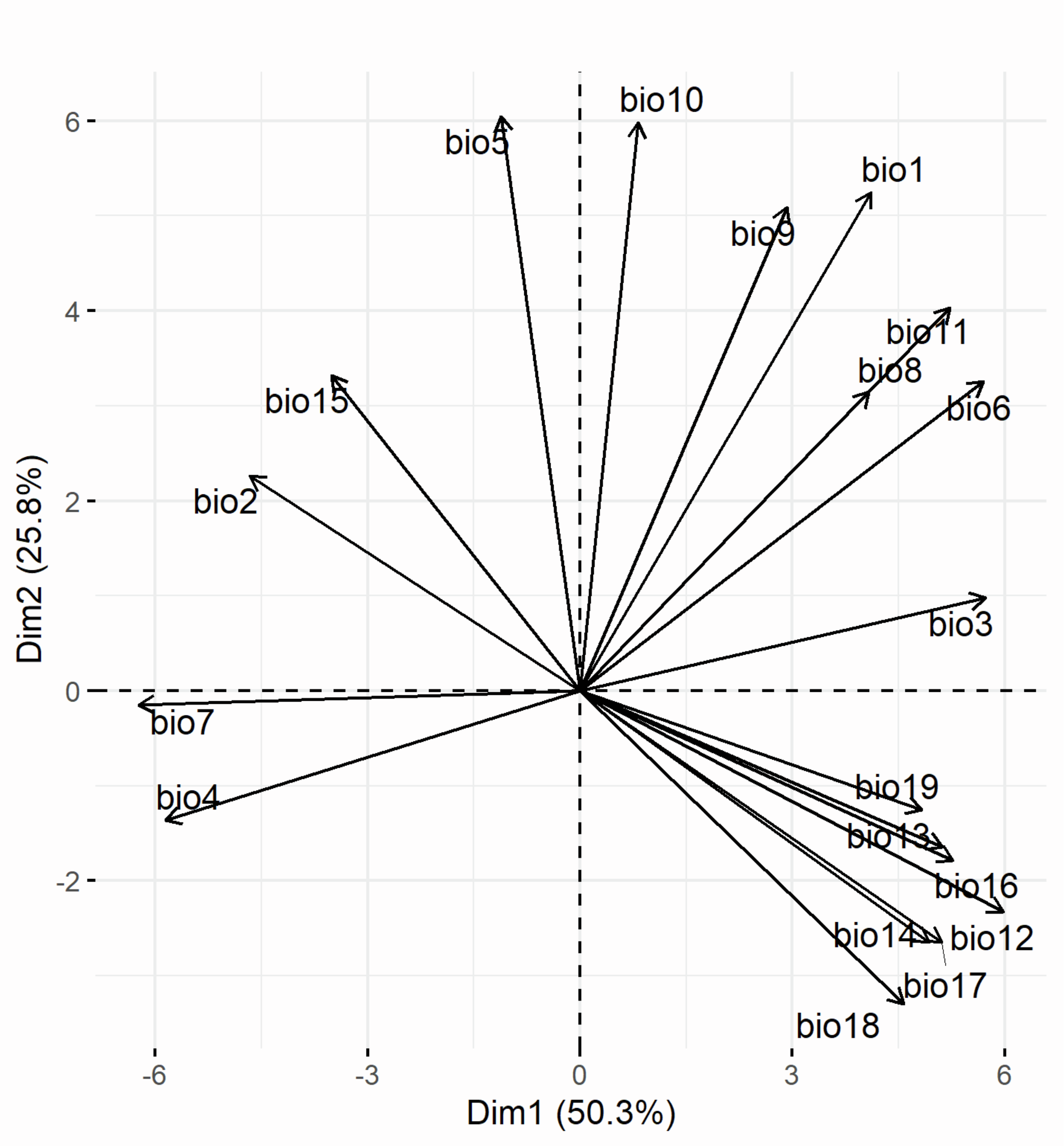

Figure S2. Principal components analysis of 19 bioclimatic variables averaged over 1970–2000. Predictor metric abbreviations are BIO1 = Annual Mean Temperature, BIO2 = Mean Diurnal Range (Mean of monthly (max temp–min temp)), BIO3 = Isothermality (BIO2/BIO7) (×100), BIO4 = Temperature Seasonality (standard deviation ×100), BIO5 = Max Temperature of Warmest Month, BIO6 = Min Temperature of Coldest Month, BIO7 = Temperature Annual Range (BIO5–BIO6), BIO8 = Mean Temperature of Wettest Quarter, BIO9 = Mean Temperature of Driest Quarter, BIO10 = Mean Temperature of Warmest Quarter, BIO11 = Mean Temperature of Coldest Quarter, BIO12 = Annual Precipitation, BIO13 = Precipitation of Wettest Month, BIO14 = Precipitation of Driest Month, BIO15 = Precipitation Seasonality (Coefficient of Variation), BIO16 = Precipitation of Wettest Quarter, BIO17 = Precipitation of Driest Quarter, BIO18 = Precipitation of Warmest Quarter, BIO19 = Precipitation of Coldest Quarter. **Related to STAR Methods.**

Table S5. Socioeconomic factors tested. **Related to STAR Methods**.

Hypothesis		Descriptions	Proxy
	Mitigation potential	Maximum annual carbon-sink potential from management, protection, and restoration actions.	Reforestation, peatland restoration, natural forest management, improved rice cultivation, grazing (optimal intensity), grazing (legumes), avoided peatland impacts, avoided coastal impacts (mangroves).
**Efforts and effectiveness**	Financing	Economically sound (sustainable) financing approaches can foster the decarbonisation of the global economy and enhance biodiversity conservation[1,2].	Development aid (US$ 2011).
	Adaptive capacity	The quality of governance is key to shaping which interventions are adopted and why. It is essential to understand how financing, implementation and governance of those interventions are important components of the nature-based solution framework[2]. Effective governance and improved social capacity will foster an adequate environment for the deployment and subsequent performance of NCS. Using nature-based thinking[3], we conceptualised the nexus between governance and NCS.	Governance readiness is a composite of political stability and non-violence, control of corruption, the rule of law, and regulatory quality.<br><br>Social adaptive readiness encapsulates social inequality, ICT infrastructure, education and innovation.
	Area-based management	Expanding protected area boundaries as part of countries' NDCs contributes to NCS into the future[4].	Protected areas coverage (PA size/km^2)
**Socioeconomic statuses**	Economic readiness	Economic growth drives environmental degradation, but a high economic level can improve environmental performance[5].	National-level gross domestic product (National GDP) [US$ 2011].
	Human population size	Human population size can drive decisions on resource allocations and challenge effective environmental performance efforts.	Population density (people/km^2).
	Food insecurity		A composite measure of (1) cereal import dependency ratio, (2) per cent of arable land equipped for irrigation, (3) value of food imports over total merchandise exports, (4) political stability and absence of violence/terrorism, (5) per capita food production variability, and (6) per capita food supply variability.

Hypothesis		Descriptions	Proxy
**Ecological attributes**	Challenging soil conditions	The quality of soils is integral to ecosystem functioning and indirectly affects land-use change.	Topsoil pH.
	Species richness	Ecological outcomes include increased plant or animal species populations, diversity of species or habitats, community composition or habitat quality[6].	The richness of threatened tetrapods, including birds, amphibians, mammals and reptiles.
	Climate variability	Spatial and temporal changes in climate can impact how plants and soil interact to facilitate ecosystem functioning and influence natural regeneration[7].	The first principal component (PC-1) of 19 bioclimatic variables averaged from 1970–2000 (Figure S2).

References

S1. Bos, M., Pressey, R.L., and Stoeckl, N. (2015). Marine conservation finance: The need for and scope of an emerging field. Ocean Coast. Manag. 114, 116–128. 10.1016/j.ocecoaman.2015.06.021.

S2. Seddon, N., Chausson, A., Berry, P., Girardin, C.A.J., Smith, A., and Turner, B. (2020). Understanding the value and limits of nature-based solutions to climate change and other global challenges. Philos. Trans. R. Soc. B Biol. Sci. 375, 20190120. 10.1098/rstb.2019.0120.

S3. Randrup, T.B., Buijs, A., Konijnendijk, C.C., and Wild, T. (2020). Moving beyond the nature-based solutions discourse: introducing nature-based thinking. Urban Ecosyst. 23, 919–926. 10.1007/s11252-020-00964-w.

S4. Melillo, J.M., Lu, X., Kicklighter, D.W., Reilly, J.M., Cai, Y., and Sokolov, A.P. (2016). Protected areas' role in climate-change mitigation. Ambio 45, 133–145. 10.1007/s13280-015-0693-1.

S5. Grossman, G.M., and Krueger, A.B. (1995). Economic growth and the environment. Q. J. Econ. 110, 353–377.

S6. Chausson, A., Turner, B., Seddon, D., Chabaneix, N., Girardin, C.A.J., Kapos, V., Key, I., Roe, D., Smith, A., Woroniecki, S., et al. (2020). Mapping the effectiveness of nature-based solutions for climate change adaptation. Glob. Chang. Biol. 26, 6134–6155. 10.1111/gcb.15310.

S7. Cook-Patton, S.C., Gopalakrishna, T., Daigneault, A., Leavitt, S.M., Platt, J., Scull, S.M., Amarjargal, O., Ellis, P.W., Griscom, B.W., McGuire, J.L., et al. (2020). Lower cost and more feasible options to restore forest cover in the contiguous United States for climate mitigation. One Earth 3, 739–752. 10.1016/j.oneear.2020.11.013.
